# Multiple Hypointense Vessels are Associated with Cognitive Impairment in Patients with Single Subcortical Infarction

**DOI:** 10.1007/s12975-023-01206-9

**Published:** 2023-12-05

**Authors:** Tang Yang, Pengfei Peng, Shuai Jiang, Yuying Yan, Yi Hu, Hang Wang, Chen Ye, Ruosu Pan, Jiayu Sun, Bo Wu

**Affiliations:** 1 Department of Neurology, West China Hospital, Sichuan University, No. 37, Guo Xue Xiang, Chengdu, 610041 China; 2 Department of Radiology, West China Hospital, Sichuan University, No. 37, Guo Xue Xiang, Chengdu, 610041 China

**Keywords:** Cortical Veins, Deep Medullary Veins, Cognitive Impairment, Vessel-wall Magnetic Resonance Imaging, Subcortical Infarction

## Abstract

We aimed to explore the relationship between multiple hypointense vessels and cognitive function in patients with single subcortical infarction (SSI) and the role of SSI with different etiological mechanisms in the above relationship. Multiple hypointense vessels were measured by the number of deep medullary veins (DMVs), DMVs score, and cortical veins (CVs) score. The Montreal Cognitive Assessment (MoCA), the Shape Trail Test (STT), and the Stroop Color and Word Test (SCWT) were assessed to evaluate cognitive function. SSI was dichotomized as branch atheromatous disease (BAD) and cerebral small vessel disease (CSVD)-related SSI by whole-brain vessel-wall magnetic resonance imaging. We included a total of 103 acute SSI patients. After adjustments were made for related risk factors of cognitive function, the SSI patients with higher DMVs score were more likely to have longer STT-B (P = 0.001) and smaller STT-B-1 min (P = 0.014), and the SSI patients with higher CVs score were more likely to have shorter STT-A (P = 0.049). In subgroup analysis, we found that the negative relationship between DMVs scores and cognitive function and the positive relationship between CVs scores and cognitive function were significantly stronger in BAD patients. We provided valuable insights into the associations between DMVs, CVs, and multi-domain cognitive impairment in SSI patients, which underscored the necessity to further study the dynamic alterations of venules and their specific influence on post-stroke cognitive impairment.

## Introduction

**Fig. 1:**
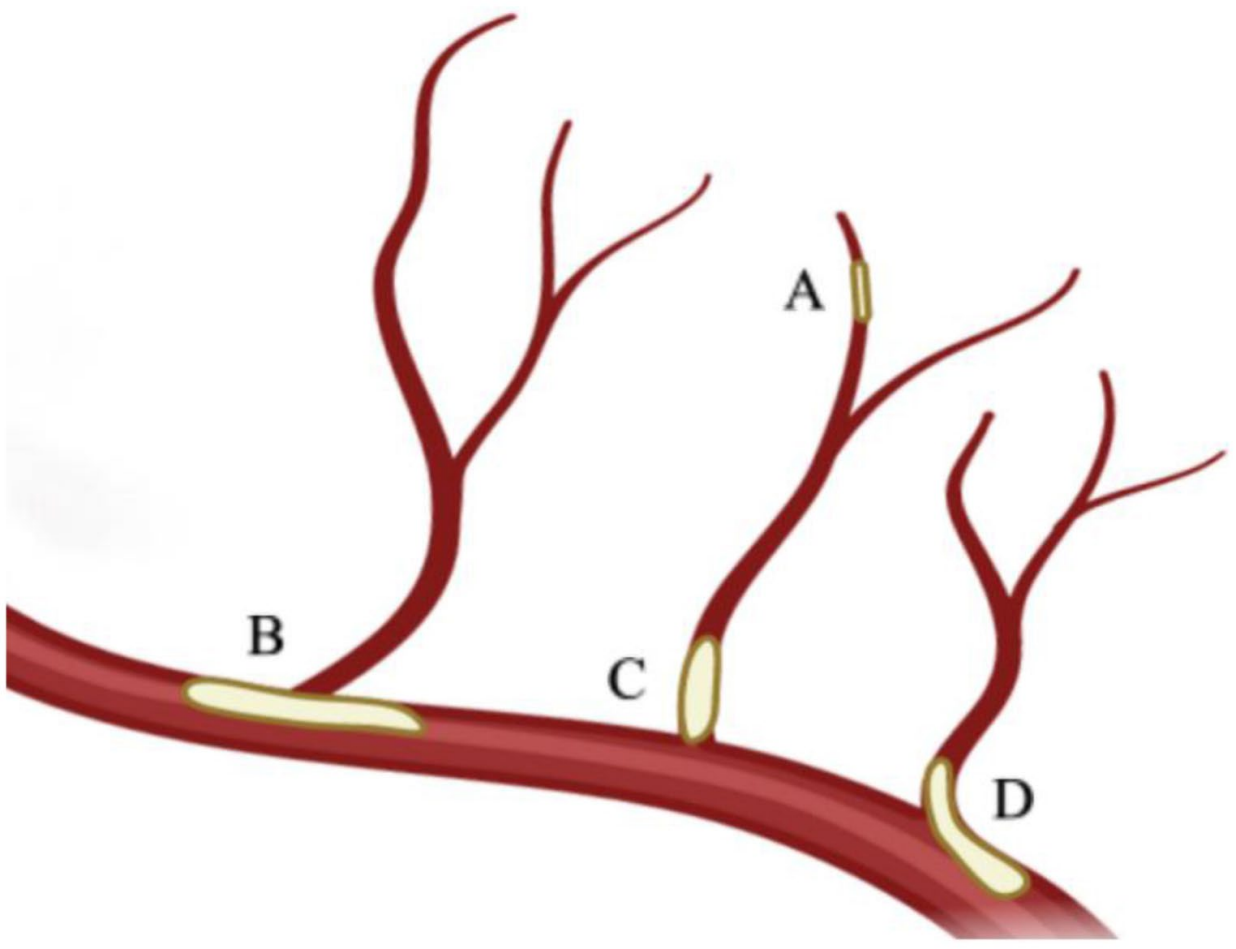
Schematic drawing showing the arterial pathologies in different etiological types of single subcortical infarction. (**A**) lipohyalinosis and fibrinoid degeneration of distal small artery; (**B**) carrier artery plaque blocking the origin of a branch artery; (**C**) obstruction at the origin of branch artery by microatheroma; (**D**) carrier artery plaque extending into the branch artery

The etiologies of single subcortical infarction (SSI) without stenotic parental artery are diverse. According to previous studies [1, 2], the two main etiological types of SSI are cerebral small vessel disease (CSVD) related SSI and branch atheromatous disease (BAD). CSVD-related SSI is characterized by lipohyalinosis and fibrinoid degeneration, so-called lacunar infarction in clinical practice. The pathology characteristic of BAD can be categorized into three conditions (Fig. 1) [2], including carrier artery plaque blocking the origin of a branch artery, carrier artery plaque extending into the branch artery, and obstruction at the origin of branch artery by microatheroma. On the other hand, about 30% of patients with lacunar infarction (including the presence of prior lacunar stroke or SVD) will have cognitive impairment in the following 4 years [3]. Moreover, we have found that BAD patients perform worse than CSVD-related SSI patients in multiple cognitive domains [4]. However, the potential risk factors that contribute to the differences in cognitive function between the two subtypes of SSI remain unclear.

Changes in draining veins after cerebral ischemia are better understood due to the application of susceptibility-weighted imaging (SWI) [5]. In acute ischemic stroke, increased concentration of deoxyhemoglobin leads to decreased signal intensity of vessels in SWI [6]. Therefore, the sign of multiple hypointense vessels may involve deep medullary veins (DMVs) and cortical veins (CVs) [7]. A recent study showed that the DMVs score is a new imaging biomarker for identifying cognitive impairment in CSVD patients [8]. Nevertheless, little is known about whether the alterations of DMVs and CVs are associated with cognitive impairment in patients with SSI, and how the alterations of DMVs and CVs affect cognitive function in SSI patients with different etiological mechanisms.

In CSVD patients, the stenosis or occlusion of veins could lead to venous hypertension and retrograde venous blood flow, and these changes might be related to the destruction of blood-brain barrier and brain white matter microstructure, and the patients might finally present with cognitive decline. Therefore, we aimed to explore the relationship between multiple hypointense vessels (measured by the number of DMVs, DMVs score, and CVs score) and cognitive function in patients with SSI. Besides, we used whole-brain vessel-wall imaging (WB-VWI) to distinguish the two subtypes of SSI [9], which is available for visualizing the lenticulostriate artery (LSA) lumen and middle cerebral artery (MCA) vessel wall in one image setting, to further investigate whether the poorer cognitive function of BAD patients compared with that of CSVD-related SSI patients is related to the number of DMVs, DMVs score, or CVs score.

## Method

### Patients

**Fig. 2:**
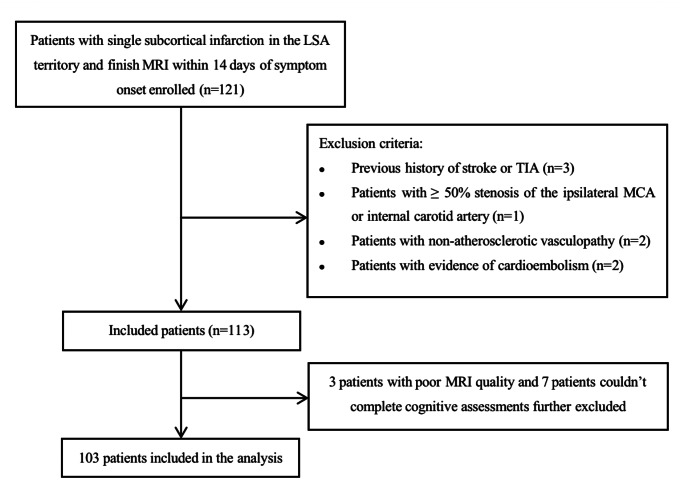
Flowchart for patient selection. LSA, lenticulostriate artery; MRI, magnetic resonance imaging; TIA, transient ischemic attacks; MCA, middle cerebral artery

We prospectively collected acute SSI patients admitted to our hospital from August 2018 to May 2022. This study was approved by the institutional review board of our hospital. Signed informed consent was obtained from all participants (or their legally authorized representatives) in the study. The inclusion criteria were: (1) SSI in the LSA territory (basal ganglia, internal capsule, and corona radiata) identified by diffusion-weighted imaging (DWI); (2) finished SWI and WB-VWI within 14 days of symptom onset. The exclusion criteria were: (1) previous history of stroke or transient ischemic attacks; (2) previous history of depression, cognitive impairment, or other cerebral pathology; (3) patients with ≥ 50% stenosis of the ipsilateral MCA or internal carotid artery confirmed by computed tomography angiography; (4) patients with non-atherosclerotic vasculopathy (e.g. dissection, vasculitis, and moyamoya disease); (5) patients with evidence of cardioembolism confirmed by transthoracic echocardiography and Holter monitoring (or 24-h electrocardiographic), such as atrial fibrillation, patent foramen ovale, valvular heart disease, infective endocarditis, and dilated cardiomyopathy; (6) patients with epileptic seizures, sepsis, decreased renal function, electrical imbalance, or other metabolic conditions that can influence the cognitive assessments. Flowchart for patient selection is shown in Fig. 2.

### Clinical Information and Cognitive Assessments

The demographic characteristics and vascular risk factors of the study patients were recorded, including age, sex, education year, smoking, drinking, hypertension, diabetes mellitus, hyperlipidemia, time from symptom onset to admission, infarct volume, and lesion location. The National Institutes of Health Stroke Scale (NIHSS) was assessed to measure the severity of neurological deficits. The Beijing version of the Montreal Cognitive Assessment (MoCA), the Shape Trail Test (STT), and the Stroop Color and Word Test (SCWT) were assessed to evaluate cognitive function during hospitalization. A detailed introduction to these cognitive scales can be seen in our previous study [4].

### Imaging Protocol

All the study patients finished magnetic resonance imaging (MRI) examinations on a research-dedicated 3.0-Tesla scanner (MAGNETOM Trio, Siemens, Erlangen, Germany) using a 32-channel head coil. The protocol included conventional T1-weighted, T2-weighted, fluid-attenuated inversion recovery imaging, DWI, SWI, WB-VWI, and Three-dimensional time-of-flight magnetic resonance angiography. Imaging parameters for SWI were TR/TE = 28/20 ms; 72 slices with a slice thickness of 2 mm; voxel size = 0.6$\times$0.6$\times$2 mm^3^; scan time = 6 min. The imaging parameters of other sequences were provided in our previous study [10].

### Classification of BAD and CSVD-related SSI

To visualize the MCA vessel wall and LSA lumen in one image setting, WB-VWI images were used to generate multi-planar reconstruction and coronal minimum intensity projection (minIP). SSI was classified into BAD (a culprit plaque positioned proximal to the LSA origin) and CSVD-related LI (the plaque positioned distal to the LSA origin or no plaque) based on WB-VWI. A demonstration of the classification method has been published previously [9].

### Assessment of CSVD MRI Markers

According to the standards for reporting vascular changes on neuroimaging (STRIVE) criteria proposed by Wardlaw et al. [11], the four MRI markers of CSVD were defined as follows: (1) Lacunes were defined as small (3-15 mm) round or ovoid subcortical lesions, of cerebrospinal fluid signal intensity on T1, T2, and fluid-attenuated inversion recovery (FLAIR), usually with a hyperintense rim on FLAIR; (2) White matter hyperintensity (WMH) was defined as abnormal hyperintensity of the deep white matter or periventricular white matter on FLAIR images. In the present study, a Fazekas score of ≥ 2 in deep white matter and/or a Fazekas score of 3 in periventricular white matter [12] were regarded as the presence of WMH; (3) Cerebral microbleeds (CMBs) were defined as homogeneous rounded lesions (2–10 mm in diameter) of signal loss on SWI; (4) Enlarged perivascular spaces (EPVSs) were defined as small (< 3 mm) dot-like or linear hyperintensities on T2-weighted imaging in the basal ganglia or centrum semiovale. The presence of moderate to severe EPVSs in our study was identified by finding > 10 EPVSs in unilateral basal ganglia. A CSVD compound score ranging from 0 to 4 was established, depending on the presence or absence (1 or 0) of each CSVD MRI marker.

### Measurement of DMVs and CVs

**Fig. 3:**
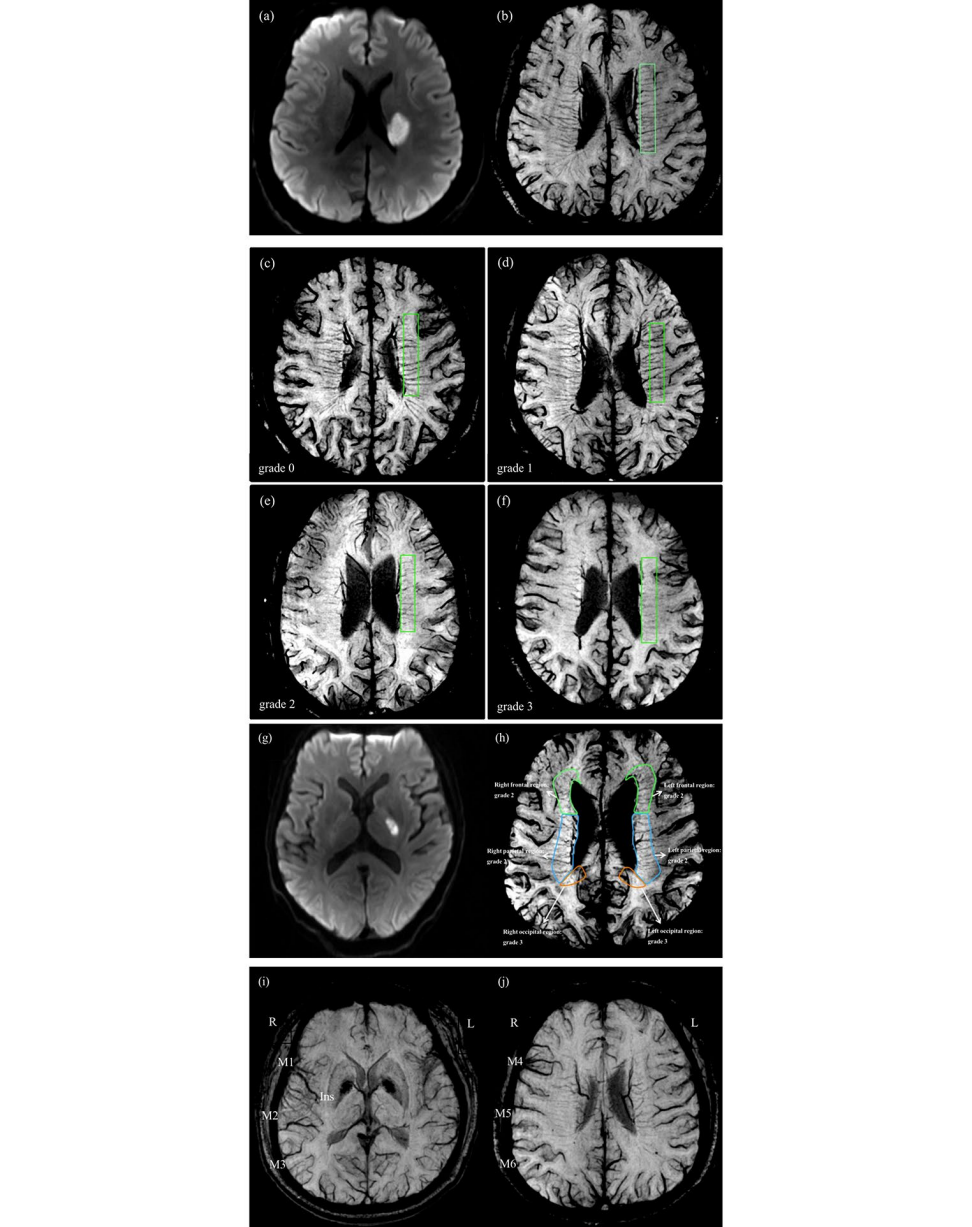
Demonstration of the measurement of multiple hypointense vessels. (**a**), (**b**): A 48-year-old male, a subcortical infarction of the left cerebral hemisphere on diffusion weighted imaging (DWI), and deep medullary veins (DMVs) counting; (**c**) to (**f**): A schematic illustration of the four-point DMVs score: Grade 0 indicates each vein is continuous and with prominent visibility; Grade 1 indicates each vein is continuous, but at least one vein with inhomogeneous signal; Grade 2 indicates at least one vein is not continuous and with faint visibility, presenting with spot-like hypointensities; Grade 3 indicates no vein is continuous; (**g**), (**h**): A 61-year-old male, a subcortical infarction of the left cerebral hemisphere on DWI, and his DMVs score is 14; (**i**), (**j**): A schematic illustration of cortical veins (CVs) score, CVs of the right cerebral hemisphere are present in more numerous or larger in the regions of M1, M3, and M4 than in the left cerebral hemisphere, and the patient’s CVs score is 3 (R = right, L = left)

To measure DMVs and CVs, the SWI phase images were processed by minIP with a slice thickness of 10 mm. We counted the number of DMVs in a region of interest of 60 mm$\times$10 mm located in periventricular white matter of the affected hemisphere for each patient (Fig. 3b) [13]. Besides, we also semiquantitatively assessed DMVs using a scoring method ranging from 0 to 18, which consisted of six regions including bilateral frontal, parietal, and occipital regions (Fig. 3h) [14]. A four-point score [8, 14] (ranging from 0 to 3) was used to evaluate DMVs of each region based on their continuity and visibility (Fig. 3c-f). Therefore, a score of 0 indicated prominent DMVs and a score of 18 indicated the absence of DMVs.

Asymmetric cortical vein (ACV) was defined qualitatively as larger or more cortical veins of the affected hemisphere compared to those of the unaffected hemisphere on SWI minIP. The draining veins of MCA territories [15] were divided into eight regions: (1) insular cortex (Ins); (2) anterior MCA cortex (M1); (3) MCA cortex lateral to the insular cortex (M2); (4) posterior MCA cortex (M3); (5) anterior MCA territories immediately superior to M1(M4); (6) lateral MCA territories immediately superior to M2 (M5); (7) posterior MCA territories immediately superior to M3 (M6); and (8) deep white matter. Each region was scored 1 if ACV was present and 0 if not. The total score of CV ranged from 0 to 7 (regions were scored except for deep white matter), a score of 0 indicated normal SWI, while a score of 7 indicated extensive dilatation of CVs (Fig. 3i and j).

### Image Analysis

Two trained investigators (TY and YYY), who were blinded to the patient’s clinical data, reviewed and analyzed all the images with commercial software (OsiriX MD, Pixmeo SARL, Bernex, Switzerland). Cohen’s kappa coefficient of the inter-rater reliability was 0.85 for classifying the SSI. Intraclass correlation coefficients of the inter-rater reliability were 0.95 for the total CSVD score, 0.78 for the number of DMVs, 0.75 for the DMVs score, and 0.80 for the CVs score.

### Statistical Analysis

Patients were dichotomized according to the median of the number of DMVs, DMVs score, or CVs score. Continuous variables were described as mean ± standard deviation (SD) or median [interquartile range (IQR)], while categorical variables were presented as frequencies and proportions. For normally distributed data, continuous variables were compared using *t*-test. For skewed data, continuous variables were compared using the Mann-Whitney *U* test. Categorical variables were compared using Chi-square or Fisher exact test. A generalized linear model was used to assess the association between multiple hypointense vessels and cognitive function in SSI patients. In generalized linear models, the covariates and factors were selected based on univariable analyses (P < 0.1) and clinical significance. In subgroup analysis, we stratified analyses by the different etiological mechanisms of SSI to assess the association between multiple hypointense vessels and cognitive function. In the generalized linear model and subgroup analysis, STT-A and STT-B were transformed using the natural logarithm to improve normality. Statistical significance was defined as two-sided P < 0.05. All statistical tests were conducted using SPSS version 26.0 (IBM, Armonk, NY, USA).

## Results

Among the 103 SSI patients, the mean age was 54.26 ± 9.715 years and 84 (81.6%) were male. The SSI patients were dichotomized at the median: (1) smaller number of DMVs group (n = 62) and bigger number of DMVs group (n = 41), median = 5; (2) low DMVs score group (n = 67) and high DMVs score group (n = 36), median = 13; (3) low CVs score group (n = 65) and high CVs score group (n = 38), median = 1. SSI patients with a bigger number of DMVs were more likely to be male (p = 0.018) and had shorter time of STT-B (p = 0.023), compared with those with a smaller number of DMVs (Table 1). SSI patients with high DMVs score had longer time of STT-B (p = 0.008) and smaller STT-B-1 min (p = 0.035), compared with those with low DMVs score (Table 1). SSI patients with high CVs score were more likely to be male (p = 0.008), smokers (p = 0.010), and drinkers (p = 0.013), compared with those with low CVs score (Table 2).

**Table 1:** Baseline characteristics in different number of DMVs and DMVs score groups

	number of DMVs ≤ 5 (n = 62)	number of DMVs > 5 (n = 41)	p	DMVs score ≤ 13 (n = 67)	DMVs score > 13 (n = 36)	p
Demographics						
Age (years)	54.61 ± 9.563	53.73 ± 10.035	0.654	54.12 ± 9.740	54.53 ± 9.799	0.840
Male sex	46 (74.2%)	38 (92.7%)	0.018	57 (85.1%)	27 (75.0%)	0.209
Education (years)	9 (9–16)	9 (9–12)	0.546	12 (9–12)	9 (9–15)	0.688
Clinical variables						
Smoking	35 (56.5%)	24 (58.5%)	0.834	39 (58.2%)	20 (55.6%)	0.795
Drinking	25 (40.3%)	21 (51.2%)	0.276	29 (43.3%)	17 (47.2%)	0.701
Hypertension	34 (54.8%)	28 (68.3%)	0.172	40 (59.7%)	22 (61.1%)	0.889
Diabetes mellitus	21 (33.9%)	11 (26.8%)	0.450	21 (31.3%)	11 (30.6%)	0.934
Hyperlipidemia	18 (29.0%)	9 (22.0%)	0.424	19 (28.4%)	8 (22.2%)	0.500
BAD	46 (74.2%)	25 (61.0%)	0.156	44 (65.7%)	27 (75.0%)	0.329
Time, onset to admission (days)	2 (1–4)	2 (1–4)	0.488	2.00 (1.00–4.00)	2.00 (1.25-4.00)	0.851
NIHSS score	4 (2–6)	2 (1–5)	0.070	4 (2–6)	3 (1–6)	0.210
Radiological data						
Infarct volume (cm^3^)	1.475 (0.7475-2.55)	1.06 (0.38–2.375)	0.087	1.21 (0.64–2.04)	1.615 (0.60–2.82)	0.571
Lesion location			0.621			0.847
Internal capsule	21 (61.8%)	13 (38.2%)		22 (62.9%)	13 (37.1%)	
Putamen and pallidum	28 (63.6%)	16 (36.4%)		30 (68.2%)	14 (31.8%)	
Other location	13 (52.0%)	12 (48.0%)		15 (62.5%)	9 (37.5%)	
≥ 1 Lacunes	26 (41.9%)	25 (61.0%)	0.059	35 (52.2%)	16 (44.4%)	0.451
≥ 1 CMBs	25 (40.3%)	18 (43.9%)	0.718	29 (43.3%)	14 (38.9%)	0.666
Moderate to severe EPVSs	37 (59.7%)	22 (53.7%)	0.546	41 (61.2%)	18 (50.0%)	0.273
WMH	14 (22.6%)	15 (36.6%)	0.122	22 (32.8%)	7 (19.4%)	0.150
Total CSVD score	1.5 (1.0–3.0)	2.0 (1.0–3.0)	0.278	3.00 (2.00–4.00)	2.75 (1.00-3.30)	0.165
Cognitive data						
MoCA score	24.50 (19.75-27.00)	24.00 (21.00–27.00)	0.824	25.00 (21.00–27.00)	23.50 (20.25-27.00)	0.546
Visuospatial/executive function	4 (2–5)	4 (3–5)	0.774	4.00 (2.00–5.00)	3.50 (2.00-4.75)	0.567
Naming	3 (2–3)	3 (3–3)	0.128	3 (3–3)	3 (2–3)	0.396
Attention	6 (5–6)	5 (5–6)	0.405	6.0 (5.0–6.0)	5.5 (5.0–6.0)	0.716
Abstraction	1.0 (1.0–2.0)	2.0 (0.5-2.0)	0.927	2 (1–2)	1 (1–2)	0.074
Language	2 (2–3)	3 (2–3)	0.675	2.0 (2.0–3.0)	2.5 (2.0–3.0)	0.854
Delayed memory	3.00 (1.00-3.25)	2.00 (1.00–4.00)	0.970	2 (1–4)	3 (2–4)	0.471
Orientation	6.0 (5.0–6.0)	6.0 (5.5-6.0)	0.298	6 (5–6)	6 (5–6)	0.649
STT-A, s	74.50 (57.75–110.50)	76.00 (53.00–89.00)	0.509	66.00 (53.00–89.00)	81.00 (62.25–118.50)	0.053
STT-B, s	194.50 (136.25-265.25)	145.00 (115.50-206.50)	0.023	153.0 (119.0-222.0)	208.0 (147.5-297.0)	0.008
STT-B-1 min	8.00 (5.75-12.00)	10.00 (8.00–13.00)	0.056	10 (8–13)	8 (5–12)	0.035
Stroop-A (time), s	32.0 (27.0–42.0)	32.0 (26.5–40.0)	0.780	32.0 (27.0–39.0)	33.0 (27.5–41.5)	0.618
Stroop-A (correct)	50 (49–50)	50 (50–50)	0.121	50 (50–50)	50 (49–50)	0.147
Stroop-B (time), s	55.00 (45.75-73.00)	60.00 (42.00-80.50)	0.511	57.00 (45.00–74.00)	59.50 (38.00-74.25)	0.931
Stroop-B (correct)	49 (46–50)	49 (45–50)	0.566	49.00 (46.00–50.00)	48.00 (45.25-50.00)	0.815
Stroop-C (time), s	101.00 (80.75-137.75)	104.00 (76.50–135.00)	0.973	103.00 (80.00-127.00)	102.50 (74.75-139.25)	0.953
Stroop-C (correct)	47.00 (43.75-49.00)	47.00 (40.50–50.00)	0.951	47.00 (43.00–49.00)	47.00 (37.25-49.00)	0.709

DMVs, deep medullary veins; BAD, branch atheromatous disease; NIHSS, National Institutes of Health Stroke Scale; CMBs, cerebral microbleeds; EPVSs, enlarged perivascular spaces; WMH, white matter hyperintensity; CSVD, cerebral small vessel disease; MoCA, Montreal Cognitive Assessment; STT, Shape Trail Test

**Table 2:** Baseline characteristics in different CVs score groups

	CVs score ≤ 1 (n = 65)	CVs score > 1 (n = 38)	P
Demographics			
Age (years)	55.620 ± 9.770	51.950 ± 9.291	0.064
Male sex	48 (73.8%)	36 (94.7%)	0.008
Education (years)	9.00 (9.00–12.00)	12.00 (9.00-15.25)	0.330
Clinical variables			
Smoking	31 (47.7%)	28 (73.7%)	0.010
Drinking	23 (35.4%)	23 (60.5%)	0.013
Hypertension	39 (60.0%)	23 (60.5%)	0.958
Diabetes mellitus	21 (32.3%)	11 (28.9%)	0.722
Hyperlipidemia	17 (26.2%)	10 (26.3%)	0.986
BAD	45 (69.2%)	26 (68.4%)	0.932
Time, onset to admission (days)	2 (1–4)	2 (1–4)	0.933
NIHSS score	3.00 (2.00–6.00)	3.00 (1.00-5.25)	0.426
Radiological data			
Infarct volume (cm^3^)	1.21 (0.675–2.735)	1.395 (0.5175–2.0175)	0.440
Lesion location			0.980
Internal capsule	21 (61.8%)	13 (38.2%)	
Putamen and pallidum	28 (63.6%)	16 (36.4%)	
Other location	16 (64.0%)	9 (36.0%)	
≥ 1 Lacunes	28 (43.1%)	23 (60.5%)	0.087
≥ 1 CMBs	25 (38.5%)	18 (47.4%)	0.376
Moderate to severe EPVSs	35 (53.8%)	24 (63.2%)	0.357
WMH	20 (30.8%)	9 (23.7%)	0.440
Total CSVD score	2 (0–3)	2 (1–3)	0.254
Cognitive data			
MoCA score	24.0 (19.0-26.5)	25.0 (22.0–28.0)	0.053
Visuospatial/executive function	4 (2–5)	4 (3–5)	0.331
Naming	3 (2–3)	3 (3–3)	0.048
Attention	6 (5–6)	5 (5–6)	0.382
Abstraction	1 (1–2)	2 (1–2)	0.004
Language	2.0 (1.5-3.0)	3.0 (2.0–3.0)	0.008
Delayed memory	2 (1–3)	3 (1–4)	0.226
Orientation	6.00 (5.00–6.00)	6.00 (5.75-6.00)	0.105
STT-A, s	83.00 (63.00-113.00)	59.00 (47.75–84.75)	0.001
STT-B, s	198.00 (133.00-265.50)	145.00 (113.75–193.50)	0.004
STT-B-1 min	8 (5–12)	11 (8–13)	0.003
Stroop-A (time), s	33.00 (29.00–43.00)	30.00 (25.75–35.25)	0.018
Stroop-A (correct)	50 (50–50)	50 (50–50)	0.866
Stroop-B (time), s	61.080 ± 22.164	58.820 ± 20.796	0.611
Stroop-B (correct)	48.0 (45.5–50.0)	49.0 (46.0–50.0)	0.269
Stroop-C (time), s	104.00 (86.50-138.50)	97.00 (72.75–134.50)	0.165
Stroop-C (correct)	47.0 (40.5–49.0)	48.0 (44.0–50.0)	0.066

CVs, cortical veins; BAD, branch atheromatous disease; NIHSS, National Institutes of Health Stroke Scale; CMBs, cerebral microbleeds; EPVSs, enlarged perivascular spaces; WMH, white matter hyperintensity; CSVD, cerebral small vessel disease; MoCA, Montreal Cognitive Assessment; STT, Shape Trail Test

### Association Between the Number of DMVs and Cognitive Function in SSI Patients

The number of DMVs was not associated with STT-B ($\beta$ coefficient, -0.102; 95% confidence interval [CI], -0.279 to 0.075; P = 0.216), after adjusting for the male sex, infarct volume, NIHSS score, and lacunes (Table 3).

**Table 3:** Associations between the number of DMVs, DMVs score, and cognitive function in SSI patients

	STT-B		STT-B-1 min	
	$\beta$ coefficient (95% CI)	P	$\beta$ coefficient (95% CI)	P
Model 1^a^	-0.102 (-0.279 to 0.075)	0.261		
Model 2^b^	0.223 (0.097–0.349)	0.001		
Model 3^c^			-1.461 (-2.627 to -0.294)	0.014

DMVs, deep medullary veins; SSI, single subcortical infarction; STT, Shape Trail Test; NIHSS, National Institutes of Health Stroke Scale; CI, confidence interval

^a^ Model 1: the association between STT-B and number of DMVs; data were adjusted for male sex, infarct volume, NIHSS score, and lacunes

^b^ Model 2: the association between STT-B and DMVs score; data were adjusted for age, education year, and NIHSS score

^c^ Model 3: the association between STT-B-1 min and DMVs score; data were adjusted for age, education year, and NIHSS score

### Association Between the DMVs Score and Cognitive Function in SSI Patients

The DMVs score was positively associated with STT-B ($\beta$ coefficient, 0.223; 95% CI, 0.097–0.349; P = 0.001) and negatively associated with STT-B-1 min ($\beta$ coefficient, -1.461; 95% CI, -2.627 to -0.294; P = 0.014), after adjusting for age, education year and NIHSS score (Table 3).

### Association Between the CVs Score and Cognitive Function in SSI Patients

When adjusting for age, male sex, smoking, drinking, and lacunes, the CVs score was negatively associated with STT-A ($\beta$ coefficient, -0.154; 95% CI, -0.307 to 0.000; P = 0.049) (Table 4).

**Table 4:** Associations between CVs score and cognitive function in SSI patients^a^

	CVs score ≤ 1		CVs score > 1	
	$\beta$ coefficient (95% CI)	P	$\beta$ coefficient (95% CI)	P
Naming	Ref		0.124 (-0.116 to 0.364)	0.310
Abstraction	Ref		0.302 (-0.008 to 0.612)	0.056
Language	Ref		0.310 (-0.043 to 0.663)	0.085
STT-A	Ref		-0.154 (-0.307 to 0.000)	0.049
STT-B	Ref		-0.093 (-0.246 to 0.059)	0.231
STT-B-1 min	Ref		1.265 (-0.108 to 2.638)	0.071
Stroop-A (time)	Ref		-2.649 (-6.546 to 1.248)	0.183

CVs, cortical veins; SSI, single subcortical infarction; STT, Shape Trail Test; CI, confidence interval

^a^ Data were adjusted for age, male sex, smoking, drinking, and lacunes

### Subgroup Analysis

In subgroup analysis, we further explored the role of SSI with different etiological mechanisms in the association between multiple hypointense vessels and cognitive function (Table 5). The effect of DMVs score on STT-B (regression coefficient, 0.241; 95%CI, 0.093–0.390; P = 0.001) and STT-B-1 min (regression coefficient, -1.520; 95%CI, -2.900 to -0.140; P = 0.031) was significantly stronger in patients with BAD. Besides, the effect of CVs score on STT-A (regression coefficient, -0.215; 95%CI, -0.391 to -0.038; P = 0.017) was also significantly stronger in patients with BAD.

**Table 5:** Association of multiple hypointense vessels with cognitive function between SSI patients with different etiological mechanisms

	BAD		CSVD-related SSI	
	Regression coefficient (95% CI)	P	Regression coefficient (95% CI)	P
Model 1^a^	0.241 (0.093–0.390)	0.001	0.154 (-0.086 to 0.394)	0.208
Model 2^b^	-1.520 (-2.900 to -0.140)	0.031	-1.190 (-3.420 to 1.040)	0.294
Model 3^c^	-0.215 (-0.391 to -0.038)	0.017	-0.006 (-0.253 to 0.265)	0.962

SSI, single subcortical infarction; BAD, branch atheromatous disease; CSVD, cerebral small vessel disease; NIHSS, National Institutes of Health Stroke Scale; CI, confidence interval; DMVs, deep medullary veins; CVs, cortical veins

^a^ Model 1: the association between STT-B and DMVs score, adjust for age, education year, and NIHSS score

^b^ Model 2: the association between STT-B-1 min and DMVs score, adjust for age, education year, and NIHSS score

^c^ Model 3: the association between STT-A and CVs score, adjust for age, male sex, smoking, drinking, and lacunes

## Discussion

In the present study, the SSI patients with discontinuous or less visible DMVs in bilateral cerebral hemisphere (higher DMVs score) were more likely to have poorer cognitive function. Besides, the SSI patients with extensive dilatation of CVs (higher CVs score) were more likely to have better cognitive function. In subgroup analysis, we found that the negative relationship between DMVs scores and cognitive function and the positive relationship between CVs scores and cognitive function were significantly stronger in BAD patients.

Arterial and venous circulation of the brain are structurally and functionally integrated. With the growing age, alterations of arteries and capillaries could damage brain function by cerebral blood flow (CBF) dysregulation, ischemia, metabolic clearance disturbance, and disruption of the blood-brain barrier [16]. Meanwhile, there is increasing evidence suggesting that changes in brain venous circulation are also important in the balance of homeostasis. Our study demonstrated that DMVs score was negatively associated with cognitive function of SSI patients, mainly in the domains of executive function and memory reflected by STT-B (the longer the time consumed for STT-B, the poorer the executive function and memory) and STT-B-1 min (the bigger the number that patient correctly connect within the first one minute, the better the executive function and memory) [17]. Consistent with the study of Xu et al., CSVD patients with higher DMVs score were more likely to have cognitive impairment [8]. There are two potential reasons why the discontinuous or less visible DMVs (manifest as an increase in the DMVs score) have a close relation with cognitive decline. From the perspective of arterial circulation, the decreasing CBF caused by aging and hypertensive arteriolosclerosis may result in hemodynamics disorder, chronic hypoperfusion, hypometabolism, reduction of oxygen extraction fraction, and eventually decreased visibility of DMVs on SWI [18]. In terms of veins, the changes in DMVs’ visibility and continuity may reflect a neurodegenerative disease called venous collagenosis, which could increase the venous pressure by venous wall thickening, venous stenosis, and even occlusion [19, 20]. In the course of the above alterations in DMVs, the CSVD burden also changed accordingly, such as leukoaraiosis associated with venous collagenosis [18], the rupture of small veins resulting in CMB [21], and cerebral microinfarct resulting from venule occlusion [22], and all of these changes might lead to cognitive impairment with SSI patients [23–26]. In our study, the median of total CSVD score in the low DMVs score group and high DMVs score group were 3 and 2.75 (Table 1), respectively, which reflected the heavy CSVD burden in SSI patients, despite the four CSVD markers did not show significant differences between groups.

We also found that CVs score was positively associated with the cognitive function of SSI patients, mainly in domains of language and attention reflected by STT-A (the longer the time consumed for STT-A, the poorer the language and attention) [17]. The two main factors for prognosis with ischemic stroke patients are collaterals and ischemia tolerance, which could compensate for ischemic injury and contribute to favorable clinical outcomes [27]. On the one hand, good collaterals and asymmetric CVs may indicate the misery perfusion of ischemic penumbra [27]. A study from Korea suggested that acute ischemic stroke patients with hypointense vessel sign on gradient echo imaging had better outcomes as this imaging marker implied ischemic penumbra [28]. Additionally, Parka et al. reported that large cerebral artery occluded patients with extensive asymmetric CVs on SWI had a good clinical outcome at 3 months [29]. On the other hand, ischemia tolerance is a neuroprotective mechanism associated with the preservation of microvascular perfusion during stroke [30]. A previous study showed that repetitive hypoxic preconditioning stimuli can induce long-term tolerance against the sustained ischemic injury of the retina [31]. Thus, ischemia tolerance might play a critical role in patients with poor collaterals but have a good clinical prognosis despite the underlying mechanism remains unclear. From our point of view, the higher the CVs score, SSI patients were more likely to have a good cognitive outcome.

In our study, the relationship between DMVs and CVs scores, and cognitive function was significantly stronger in patients with BAD than those with CSVD-related SSI. The infarctions were hypothesized to be larger in BAD than in CSVD-related SSI based on the vascular lesions of BAD being located proximally along the perforator artery in comparison to those of CSVD-related SSI [32]. In BAD patients, the bigger subcortical ischemic lesions might affect more DMVs located in the periventricular white matter and more strategic regions, leading to more serious cognitive decline. Besides, with the progression of atherosclerosis, BAD patients might have better ischemia tolerance due to some transient and nonfatal ischemic insults before the fatal ischemic attack, and a timely treatment to save penumbra tissue could contribute to a favorable cognitive outcome. However, the above hypothesis needs to be confirmed in further research.

There are some limitations that merit consideration. The DMVs and CVs scores were qualitatively but not quantitatively assessed, further study should use quantitative measures (e.g. quantitative susceptibility mapping) for accurate evaluation. Follow-up data on SWI and cognitive function were not provided in the present study, hence some of the results might lack convincing explanations. The study included a high proportion of male patients, and we need to enroll more female patients in future studies to confirm the reliability of our conclusions. This is a cohort study in a single center, our findings need to be confirmed in other samples.

In conclusion, we provided valuable insights into the associations between DMVs, CVs, and multi-domain cognitive impairment in SSI patients, and some of their associations were more pronounced in BAD patients. Our findings underscore the necessity to further study the dynamic alterations of venules and their specific influence on post-stroke cognitive impairment.

## Data Availability

The data that support the findings of this study are available from the corresponding author upon reasonable request.

## References

[1] Fisher CM. Lacunes: small, deep cerebral infarcts. Neurology. 1965;15:774–84. 14315302 10.1212/wnl.15.8.774

[2] Caplan LR. Intracranial branch atheromatous Disease: a neglected, understudied, and underused concept. Neurology. 1989;39:1246–50. 2671793 10.1212/wnl.39.9.1246

[3] Makin SDJ, Turpin S, Dennis MS, Wardlaw JM. Cognitive impairment after lacunar Stroke: systematic review and meta-analysis of incidence, prevalence and comparison with other Stroke subtypes. J Neurol Neurosurg Psychiatry. 2013;84:893–900. 23457225 10.1136/jnnp-2012-303645 PMC3717603

[4] Yang T, Deng Q, Jiang S, Yan Y-Y, Yuan Y, Wu S-M, et al. Cognitive impairment in two subtypes of a single subcortical infarction. Chin Med J. 2021;134:2992–8. 34908257 10.1097/CM9.0000000000001938 PMC8710315

[5] Jensen-Kondering U, Böhm R. Asymmetrically hypointense veins on T2*w imaging and susceptibility-weighted imaging in ischemic Stroke. World J Radiol. 2013;5:156–65. 23671751 10.4329/wjr.v5.i4.156 PMC3647207

[6] Kesavadas C, Santhosh K, Thomas B. Susceptibility weighted imaging in cerebral hypoperfusion—can we predict increased oxygen extraction fraction? Neuroradiology. 2010;52:1047–54. 20567811 10.1007/s00234-010-0733-2

[7] del Poggio A, Godi C, Calloni SF, Ragusi M, Iadanza A, Falini A, et al. Multiple hypointense veins on susceptibility weighted imaging as a promising biomarker of impaired cerebral hemodynamics in chronic steno-occlusive Disease: a multiparametric MRI study. Neuroradiology. 2022;64:2235–43. 35699773 10.1007/s00234-022-02994-x

[8] Xu Z, Li F, Xing D, Song H, Chen J, Duan Y, et al. A novel imaging biomarker for cerebral small vessel Disease associated with cognitive impairment: the deep-medullary-veins score. Front Aging Neurosci. 2021;13:720481. 34759812 10.3389/fnagi.2021.720481 PMC8572877

[9] Jiang S, Cao T, Yan Y, Yang T, Yuan Y, Deng Q, et al. Lenticulostriate artery combined with neuroimaging markers of cerebral small vessel Disease differentiate the pathogenesis of recent subcortical infarction. J Cereb Blood Flow Metab. 2021;41:2105–15. 33563077 10.1177/0271678X21992622 PMC8327122

[10] Jiang S, Yan Y, Yang T, Zhu Q, Wang C, Bai X, et al. Plaque distribution correlates with morphology of lenticulostriate arteries in single subcortical infarctions. Stroke. 2020;51:2801–9. 32757756 10.1161/STROKEAHA.120.030215 PMC7447184

[11] Wardlaw JM, Smith EE, Biessels GJ, Cordonnier C, Fazekas F, Frayne R, et al. Neuroimaging standards for research into small vessel Disease and its contribution to ageing and neurodegeneration. Lancet Neurol. 2013;12:822–38. 23867200 10.1016/S1474-4422(13)70124-8 PMC3714437

[12] Staals J, Makin SDJ, Doubal FN, Dennis MS, Wardlaw JM. Stroke subtype, vascular risk factors, and total MRI brain small-vessel Disease burden. Neurology. 2014;83:1228–34. 25165388 10.1212/WNL.0000000000000837 PMC4180484

[13] Ao D-H, Zhang D-D, Zhai F-F, Zhang J-T, Han F, Li M-L, et al. Brain deep medullary veins on 3-T MRI in a population-based cohort. J Cereb Blood Flow Metab. 2021;41:561–8. 32312169 10.1177/0271678X20918467 PMC7922755

[14] Zhang R, Zhou Y, Yan S, Zhong G, Liu C, Jiaerken Y, et al. A brain region-based deep medullary veins visual score on susceptibility weighted imaging. Front Aging Neurosci. 2017;9:269. 28848426 10.3389/fnagi.2017.00269 PMC5550668

[15] Park M-G, Yang T-I, Oh S-J, Baik SK, Kang YH, Park K-P. Multiple hypointense vessels on susceptibility-weighted imaging in acute ischemic Stroke: surrogate marker of oxygen extraction fraction in penumbra? Cerebrovasc Dis. 2014;38:254–61. 25401484 10.1159/000367709

[16] Fulop GA, Tarantini S, Yabluchanskiy A, Molnar A, Prodan CI, Kiss T, et al. Role of age-related alterations of the cerebral venous circulation in the pathogenesis of vascular cognitive impairment. Am J Physiol Heart Circ Physiol. 2019;316:H1124–40. 30848677 10.1152/ajpheart.00776.2018 PMC6580383

[17] Zhao Q, Guo Q, Li F, Zhou Y, Wang B, Hong Z. The shape trail test: application of a new variant of the trail making test. PLoS ONE. 2013;8:e57333. 23437370 10.1371/journal.pone.0057333 PMC3577727

[18] Xu Z, Li F, Wang B, Xing D, Pei Y, Yang B, et al. New insights in addressing cerebral small vessel Disease: association with the deep medullary veins. Front Aging Neurosci. 2020;12:597799. 33335483 10.3389/fnagi.2020.597799 PMC7736107

[19] Moody DM, Brown WR, Challa VR, Anderson RL. Periventricular venous collagenosis: association with leukoaraiosis. Radiology. 1995;194:469–76. 7824728 10.1148/radiology.194.2.7824728

[20] Keith J, Gao F, Noor R, Kiss A, Balasubramaniam G, Au K, et al. Collagenosis of the deep medullary veins: an underrecognized pathologic correlate of white matter hyperintensities and periventricular infarction? J Neuropathology Experimental Neurol. 2017;76:299–312. 10.1093/jnen/nlx009 28431180

[21] Ungvari Z, Tarantini S, Kirkpatrick AC, Csiszar A, Prodan CI. Cerebral microhemorrhages: mechanisms, consequences, and prevention. Am J Physiol Heart Circ Physiol. 2017;312:H1128–43. 28314762 10.1152/ajpheart.00780.2016 PMC5495931

[22] Hartmann DA, Hyacinth HI, Liao F, Shih AY. Does pathology of small venules contribute to cerebral microinfarcts and Dementia? J Neurochem. 2018;144:517–26. 28950410 10.1111/jnc.14228 PMC5869083

[23] Xu X, Lau KK, Wong YK, Mak HKF, Hui ES. The effect of the total small vessel Disease burden on the structural brain network. Sci Rep. 2018;8:7442. 29748646 10.1038/s41598-018-25917-4 PMC5945601

[24] Werring DJ. Cognitive dysfunction in patients with cerebral microbleeds on T2*-weighted gradient-echo MRI. Brain. 2004;127:2265–75. 15282216 10.1093/brain/awh253

[25] Smith EE, Schneider JA, Wardlaw JM, Greenberg SM. Cerebral microinfarcts: the invisible lesions. Lancet Neurol. 2012;11:272–82. 22341035 10.1016/S1474-4422(11)70307-6 PMC3359329

[26] van Veluw SJ, Shih AY, Smith EE, Chen C, Schneider JA, Wardlaw JM, et al. Detection, risk factors, and functional consequences of cerebral microinfarcts. Lancet Neurol. 2017;16:730–40. 28716371 10.1016/S1474-4422(17)30196-5 PMC5861500

[27] Xu Z, Duan Y, Yang B, Huang X, Pei Y, Li X. Asymmetric deep medullary veins in patients with occlusion of a large cerebral artery: association with cortical veins, leptomeningeal collaterals, and prognosis. Front Neurol. 2019;10:1292. 31866937 10.3389/fneur.2019.01292 PMC6906174

[28] Seo S, Oh H, Park T, Kim G, Chung C, Lee K. Therapeutic implications of hypointense vessel signs on gradient echo imaging in acute ischemic Stroke. Joint World Congress on Stroke; 26–29 October 2006; Cape Town, South Africa. Int J Stroke. 2006;1(Suppl s1):162–3.

[29] Park M-G, Yeom JA, Baik SK, Park K-P. Total mismatch of diffusion-weighted imaging and susceptibility-weighted imaging in patients with acute cerebral ischemia. J Neuroradiol. 2017;44:308–12. 28579039 10.1016/j.neurad.2017.04.002

[30] Dawson DA, Furuya K, Gotoh J, Nakao Y, Hallenbeck JM. Cerebrovascular hemodynamics and ischemic tolerance: lipopolysaccharide-induced resistance to focal cerebral ischemia is not due to changes in severity of the initial ischemic insult, but is associated with preservation of microvascular perfusion. J Cereb Blood Flow Metab. 1999;19:616–23. 10366191 10.1097/00004647-199906000-00004

[31] Zhu Y, Zhang Y, Ojwang BA, Brantley MA, Gidday JM. Long-term tolerance to retinal ischemia by repetitive hypoxic preconditioning: role of HIF-1alpha and heme oxygenase-1. Invest Ophthalmol Vis Sci. 2007;48:1735. 17389506 10.1167/iovs.06-1037

[32] Nah H-W, Kang D-W, Kwon SU, Kim JS. Diversity of single small subcortical infarctions according to infarct location and parent artery Disease: analysis of indicators for small vessel Disease and Atherosclerosis. Stroke. 2010;41:2822–7. 20966406 10.1161/STROKEAHA.110.599464

